# Optimization Model of Mathematics Instructional Mode Based on Deep Learning Algorithm

**DOI:** 10.1155/2022/1817990

**Published:** 2022-07-04

**Authors:** Rui Liu

**Affiliations:** Science Teaching Department, Zhengzhou Preschool Education College, Zhengzhou 450000, China

## Abstract

This paper proposes corresponding teaching methods and instructional modes based on predecessors' research on mathematics instructional mode and the current state of mathematics teaching. In addition, this paper constructs a teaching evaluation model based on DL algorithm based on an in-depth study of DL-related theories in order to accurately and scientifically analyze the problems that exist in mathematics teaching. This paper constructs an instructional quality evaluation index system based on rationality and fairness, and uses the BPNN evaluation model to train and study a set of instructional quality data. Finally, the experimental results show that this system has a high level of stability, with a 96.37 percent stability rate and a 95.42 percent evaluation accuracy rate. The results of this paper's evaluation of the mathematical instructional quality model are objective and reasonable. It can accurately assess instructional quality while also assessing problems in the teaching process based on the instructional quality scores and making reasonable recommendations for teaching improvement based on the weak links in the teaching process. It has the potential to provide a workable system for assessing instructional quality.

## 1. Introduction

The modernization of education necessitates the improvement of instructional quality [1]. Many teachers use the boring lecturing classroom mode in the traditional mathematics classroom, resulting in a serious and boring classroom atmosphere. Students are effectively shackled as a result of this, and their ability to learn in class suffers as a result. We can optimize the mathematics instructional mode to change the above teaching phenomenon. Mathematics instructional mode optimization can effectively deepen students' understanding of mathematics. Teachers should constantly optimize the mathematical model based on students' learning situations and cognitive characteristics in order for students to comprehend and master the theoretical knowledge and content of mathematics from multiple angles and dimensions during actual teaching. The new curriculum reform emphasizes establishing students' dominance in classroom instruction. This necessitates teachers completely altering the traditional model and establishing a new teacher-student relationship. In general, the optimization of a mathematical model entails the following components: language category optimization, language syntax optimization, and language logic optimization. The optimization of classroom instructional mode and the construction of effective mathematics classrooms are of great teaching and practical importance in today's environment.

The scientificity, rationality, and timeliness of instructional quality evaluation play a key role in the optimization of instructional mode. However, the limitations of the traditional instructional quality evaluation method itself make it controversial. Therefore, it is of practical significance to establish a scientific and reasonable instructional quality evaluation model to evaluate mathematics instructional quality. Deep learning (DL) [2–4] is an extension of the research field of machine learning [5–7] and an effective way to realize artificial intelligence. Due to the rapid development of DL, NN (neural network) [2, 8] in DL has also been well developed, and its effect has been well verified in many network models. NN is a complex calculation method to simulate the neuron and neuron connection structure of human brain. NN technology is mainly based on the workflow of human nerves and uses human nerves to process related contents for calculation. BPNN (backpropagation neural network) is a three-layer feedforward hierarchical network composed of input layer, hidden layer, and output layer. Its learning process consists of two processes: forward propagation of information and backward propagation of errors. BPNN has the advantages of strong generalization and fault tolerance, but it also has some shortcomings. Genetic algorithm is an algorithm that refers to and simulates biological genetic mechanism and natural selection. Its basic idea is to simulate the evolution process of population. After three genetic operations, selection, crossover, and mutation, the population is constantly updated, and the excellent degree of the population is constantly enhanced, approaching the global optimal solution. Aiming at the shortcomings of BPNN, genetic algorithm can be used to improve it. This paper mainly studies the optimization model of mathematics instructional mode based on DL algorithm, and its innovation lies in: (1) this paper improves the existing evaluation index system and constructs a new instructional quality evaluation index system based on rationality and fairness. To train and study the instructional quality data, the BPNN evaluation model is used. After demonstration, the instructional quality evaluation index system in this paper is scientific, objective, and reasonable. (2) This paper improves the problem that extracting principal components from the correlation coefficient matrix of indexes cannot reflect the difference information of the variation degree of each index by introducing the basic principle of principal component analysis. Because BPNN is highly dependent on its initial weights and thresholds, a genetic algorithm is used to optimize BPNN's initial weights and thresholds in order to improve the prediction accuracy and convergence speed of instructional quality evaluation results. The experiment shows that this system can be used as a benchmark for evaluating instructional quality and making decisions.

## 2. Related Work

Based on the content of the instructional quality evaluation index system, Sergis et al. constructed a convolutional neural network instructional quality evaluation model based on the convolutional neural network learning expert instructional quality evaluation samples [9]. Maulana et al. mainly considered different disciplines and majors, set up different evaluation index projects, and established different evaluation index systems. And we use the powerful functions of the MATLAB toolbox to establish a BP neural network, train the network, test the network, and finally analyze the experimental results [10]. Choi and Kim et al. pointed out that under the current background, more and more emphasis is placed on strengthening the interaction between teachers and students, and the main form of interaction is the “dialogue” in the classroom. And we summarize the effective instructional mode of mathematics around “dialogue” [11, 12]. Starting from a brief analysis of the optimization of classroom instructional mode, Petraki E analyzed the main points of constructing an effective mathematics classroom after expounding the problems existing in today's mathematics classroom [13]. Onozawa M uses a deep learning algorithm to design an instructional quality evaluation system, which intelligently evaluates the instructional quality and puts forward rationalization suggestions [14]. Aiming at the problems existing in the quality evaluation of NC bilingual teaching courses, Liu et al. tried to use the analytic hierarchy process to carry out exploratory analysis, thus providing a scientific case basis for the evaluation of bilingual course instructional quality [15]. Northrup et al. believed that improving the pertinence of homework is one of the core contents of constructing an effective mathematics classroom. While intensifying in-class training in mathematics and improving the effectiveness of feedback correction, we must pay attention to the efficiency of mathematics after-school homework training [16, 17]. Ottmar et al. expounded the design idea and implementation method of neural network for instructional quality evaluation, introduced and analyzed the traditional instructional quality evaluation method, and trained and tested the designed neural network for instructional quality evaluation [18].

This paper proposes corresponding teaching methods and instructional modes based on predecessors' research on mathematics instructional mode and the current state of mathematics teaching. Furthermore, this paper constructs a teaching evaluation model based on DL algorithm based on an in-depth study of DL-related theories. The optimization of the mathematics instructional mode is carried out based on the evaluation of the teaching evaluation model. In this paper, a rich basic dataset of teaching evaluation has been formed through the evaluation of teaching experts, teachers' peers, and students. The normalization formula is used to process the input samples. Furthermore, because BPNN is so reliant on its initial weights and thresholds, this paper employs a genetic algorithm to optimize BPNN's initial weights and thresholds and reduce the time it takes for BPNN to find the weights and thresholds that satisfy the training termination conditions, in order to improve NN's instructional quality evaluation results' prediction accuracy and convergence speed.

## 3. Methodology

### 3.1. Optimizing Classroom Instructional Mode and Constructing Efficient Mathematics Classroom

Math textbooks for each stage are also designed according to students' knowledge base, cognitive level, and thinking ability, as there are high expectations for mathematics students' thinking ability. However, many teachers adopt the tedious lecture-based classroom mode during the actual teaching process, resulting in a serious and monotonous classroom environment [19]. We can improve the mathematics instructional mode to change the above-mentioned teaching phenomenon. Students' understanding of mathematics can be effectively deepened by optimizing mathematics instructional mode [20]. The term “dialogue” is used in class to describe how teachers and students communicate. The amount of “dialogue” and its effect on the classroom atmosphere and instructional quality are both directly determined by the amount of “dialogue.” An effective dialogue instructional mode can help students break free from the constraints of their thinking, causing them to think and express more actively, and thus promoting the development of independent thinking abilities. Because mathematical models are all expressed in mathematical languages, they must be transformed using different mathematical languages. In order to successfully transform mathematical languages, students must master a variety of mathematical languages, including geometric language, algebraic language, and symbolic language. Therefore, in mathematics teaching, teachers should pay attention to the training of students' mutual conversion between different languages when changing “numbers” into “shapes” through the equivalent exchange of mathematical model languages. Besides, the guiding ideology of optimizing classroom instructional mode also includes the corresponding concept of “efficient classroom.” The main contents of this concept include teaching preparation, teaching process, training and testing, after-class counseling, teaching evaluation, and other aspects of different courses. Therefore, on this basis, the optimization of classroom instructional mode can be better applied.

First of all, in order to realize the correct and efficient conversion between mathematical languages, teachers must help students establish a correct mathematical language structure. Usually, mathematical models are based on mathematical concepts, which are connected by verbs. The main reasons for their complexity are the order relationship, hierarchical relationship, and logical relationship in the structure of mathematical models. Therefore, in order to correctly grasp the mathematical model, it is necessary to correctly sort out the structural relationship between mathematical concepts to ensure the correct transformation of mathematical model. Furthermore, teachers must teach students how to ask questions in class; that is, the questions posed must be of sufficient inquiry value, conform to classroom teaching content, and have a clear purpose. Students will have a better understanding of the meaning of asking questions as a result of the increased teaching time, and they will be able to grasp the points that are appropriate for asking questions more accurately and master effective questioning methods, resulting in a better awareness of questions. This not only is beneficial for creating a productive classroom but also allows students to improve their mathematical inquiry learning abilities. At the same time, the most important thing is to cultivate students' interest in mathematics. Students' interest in mathematics learning is influenced not only by the number of mathematical concepts and formulas taught in class by mathematics teachers but also by the number of classic questions explained and analyzed in exercise classes, as well as other factors. Mathematics teachers must not only have good mathematics knowledge, teaching skills, and teaching process to improve students' interest in mathematics learning; they must also have better mathematics teaching methods, mathematics teaching emotions, mathematics teaching attitudes, and mathematics teaching values.

Teachers should master the effective teaching method of question situation and avoid the traditional acceptance method of asking questions. We should not only attach importance to the results but also to the whole process of students' participation in classroom interaction. The effective construction of mathematics classroom needs the effective support of corresponding points. This is mainly reflected in the effective preparation before class, the reasonable improvement of classroom teaching efficiency, the emphasis on promoting students' cooperative learning, and the pertinence of homework after class. In classroom teaching, teachers can realize the simplification of mathematical problems by flexibly changing mathematical thinking patterns. Through the rational application of the above strategies, students can successfully realize the transformation between mathematical models. This method can get better results in the application of trigonometric function, geometry, and complex number knowledge. In the process of inquiry learning, teachers do not instill knowledge stiffly, but let students actively participate in knowledge exploration, and naturally digest and absorb knowledge in the process of exploration. Therefore, it can promote the effective improvement of mathematics classroom instructional quality and cultivate students' interest in mathematics invisibly. At the same time, the effective cooperative learning among students can play a very good role in promoting the creation and optimization of teaching links. The exploration of effective mathematics instructional mode cannot be separated from the joint efforts of students and teachers. Finally, in order to promote the continuous improvement of classroom instructional quality, teachers should also organize students to carry out classroom teaching and evaluation of learning situation, so as to better find problems and put forward improvement measures. For some good places, it is necessary to keep them.

### 3.2. Application of DL Algorithm in Instructional Quality Evaluation

**Figure 1:**
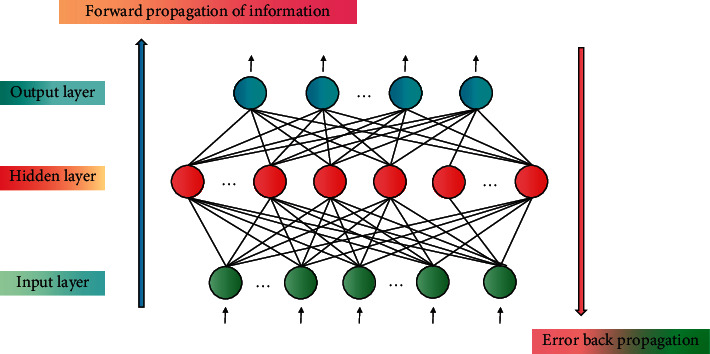
Three-layer BPNN model.

It is an extension of the research field of machine learning and an effective way to realize artificial intelligence [21]. As a new technology, DL has opened up a new way for pattern recognition [22], nonlinear classification [23], artificial intelligence, and other research with its basic characteristics such as nonlinear mapping, learning classification, and real-time optimization. It involves biology, electronics, computer, mathematics, physics, and other disciplines, and has a wide application prospect. Due to the rapid development of DL, NN in the field of DL has also been well developed, and its effect has been well verified in many network models. NN is a complex calculation method to simulate the neuron and neuron connection structure of human brain. NN technology is mainly based on the workflow of human nerves and uses human nerves to process related contents for calculation. Because of the parallelism of NN, it is very effective for solving combinatorial optimization problems. From the point of view of automatic control, NN can be regarded as a high-dimensional nonlinear dynamic system. Neurons are the processing units in this system. This system has many inputs and many outputs, and we regard its input-output relationship as a mapping from input to output. Therefore, it is reasonable to apply NN to the design of instructional quality model. The three-layer BPNN model is shown in Figure 1.

BPNN is an iterative algorithm consisting of two processes: forward propagation of input vector and backward propagation of error. The algorithm is divided into two stages: ① forward propagation of input vector. ② Backpropagation of error. BPNN has the following advantages: ① BPNN is suitable for solving problems with complex internal mechanisms. ② It can process information in parallel. ③ It can abstract the relevant laws from the samples. ④ It has a strong self-learning function. ⑤ For the input samples in BPNN, there may be errors with large errors. But BPNN still has some limitations. For example, it is easy to fall into the local optimum, the recognition accuracy is easily affected, there is no fixed basis for setting network parameters, there is a tendency to forget the old samples when learning new ones during training, and the generalization ability is poor. Based on DL algorithm, this paper constructs an instructional quality evaluation model. For an instructional quality evaluation system, it can be regarded as a mapping from input to output. NN's self-learning ability, that is, the plasticity of mapping, enables it to simulate the required mapping relationship through training, thus replacing domain experts to automatically evaluate the evaluation object.

### 3.3. Construction of the Teaching Evaluation Model Based on DL

**Figure 2:**
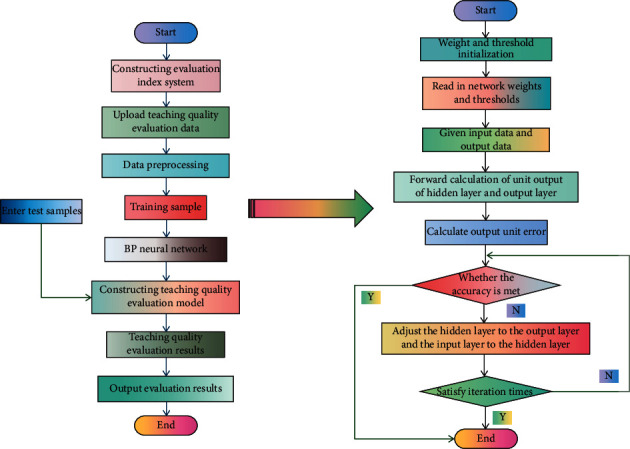
DL-based instructional quality evaluation model and algorithm flow.

Instructional quality evaluation system is an evaluation of teachers' phased teaching effect, which provides favorable analysis basis for mastering teachers' teaching ability and improving instructional quality. When evaluating the instructional quality, we should adhere to an objective, fair, and rational attitude. We should not speculate subjectively or mix personal feelings, and we should be ideological, scientific, and feasible. The establishment of a reasonable model of teacher's teaching evaluation system can not only enable teachers to revise and improve the teaching plan according to the teaching effect in time but also help the school to understand the implementation of teachers' teaching tasks and make macro-control. More importantly, a perfect evaluation system is of great significance to the establishment of a teaching evaluation model for teachers in colleges and universities. The instructional quality evaluation system based on DL algorithm is designed based on B/S mode. The advantages of designing the instructional quality evaluation system based on B/S mode are as follows: it is convenient for different types of users to operate, the online evaluation can be completed in a short time, and the system maintenance is convenient. Client, application unit, and database are three important components of the system. According to the experience of this article, we try to use an implicit layer first. The DL-based instructional quality evaluation model and algorithm flow are shown in Figure 2.

The samples used in data analysis frequently contain a large number of variables, and adding more variables increases the complexity of the analysis problem. The information contained in these principal components should be required to be independent of one another in order for them to not overlap. The main goal of principal component analysis (PCA) is to reduce the dimensions of variables and simplify problems by retaining the most important information from the original variables, making it easier to understand the main contradictions when studying complex problems. The function of DL is to learn the inherent law and representation level of sample data. The BPNN model in the DL algorithm is used to evaluate instructional quality when designing the instructional quality evaluation system. The evaluation method is a nonlinear problem because there are many uncertain and complex factors in the evaluation process. BPNN has demonstrated its unique advantages in solving such problems by having a strong adaptive learning ability that can approximate any function. The steps of BPNN learning rules are as follows: the output of the *i* neuron in the hidden layer is as follows:

(1) $$\begin{array}{c} a1_{i}=f1\left(\sum_{j=1}^{r}wl_{ij}p_{j}+b1_{i}\right),\;i=1,2,3,\ldots ,s1. \end{array}$$

The output of the *k*th neuron in the output layer is as follows:

(2) $$\begin{array}{c} a2_{k}=f2\left(\sum_{i=1}^{s1}w2_{ki}a1_{i}+b2_{i}\right),\;k=1,2,3,\ldots ,s2. \end{array}$$

We define the error function as follows:

(3) $$\begin{array}{c} E\left(W,B\right)=\frac{1}{2}\sum_{k=1}^{s2}{\left(t_{k}-a2_{k}\right)}^{2}. \end{array}$$

We assume that the evaluation indicators of the evaluation system are *N*, the result grades are *M*, and the classification of the total indicators is divided into *L* categories; the evaluation scores of each indicator can be used as the input of the scoring comprehensive network, which is expressed as follows:

(4) $$\begin{array}{c} X={\left[x_{11}<x_{1j}<x_{1m}<x_{i1}<x_{ij}<x_{im}<x_{n1}<x_{nj}<x_{nm}\right]}^{T}. \end{array}$$

The rating coefficient of the evaluation result is taken as the output of the scoring comprehensive network, which is expressed as follows:

(5) $$\begin{array}{c} K={\left[k_{1}k_{2}<k_{j}<k_{m}\right]}^{T}. \end{array}$$

The importance coefficient of each index is connected by the synaptic weight matrix, which is expressed as follows:

(6) $$\begin{array}{c} W={\left[W_{1}W_{2}<W_{j}<W_{m}\right]}^{T}. \end{array}$$

In this paper, roulette is adopted. First, the fitness value of a certain generation of individuals, namely, BPNN weight and threshold, is calculated. Then, we calculate the proportion of the fitness value in the total fitness value, that is, the probability of the individual being selected in the selection process. The calculation of the selection probability is shown in the following formula:

(7) $$\begin{array}{c} z\left(a_{j}\right)=\frac{f\left(a_{j}\right)}{\sum_{j=1}^{d}f\left(a_{j}\right)}. \end{array}$$

Among them, *a*_*j*_ represents an individual in the group, *z*(*a*_*j*_) is the probability that *a*_*j*_ is selected, *f*(*a*_*j*_) is the fitness value of individual *a*_*j*_, and *d* is the population size. After calculating the probability of each individual being selected, it is necessary to determine whether the selected individual can continue to be inherited to the next generation of individuals according to the size of the cumulative probability. The calculation formula is as shown below.

(8) $$\begin{array}{c} q\left(a_{k}\right)=\sum_{j=1}^{k}z\left(a_{j}\right). \end{array}$$

Among them, *q*(*a*_*k*_) is the cumulative probability of individual *a*_*k*_, and then, a new population of the next generation is obtained through genetic manipulation, the fitness value is judged, and the above steps are repeated until the population is stable. The action function of each neuron is taken as a linear function:

(9) $$\begin{array}{c} F\left(S_{j}\right)=S_{j}. \end{array}$$

The output of the node at this point is as follows:

(10) $$\begin{array}{c} K_{j}=S_{j}=W_{j}^{T}X. \end{array}$$

In this paper, the average method is used for dimensionless processing, which can extract more original information with less principal components, thus reducing the workload and making the solution to the problem more perfect, more accurate, and more comprehensive. Valuable data such as instructional quality evaluation index data, evaluation subject, and object data are stored in the database. The evaluation is divided into two aspects: index scoring and grading and classification. Therefore, two networks can be used for processing, and one network can store and memorize the importance coefficients of each index and synthesize the scores, which is called comprehensive network. The other one classifies the index scores linearly or nonlinearly, so as to obtain the final evaluation result of the total index, which is called classification network. In the process of evaluating the instructional quality, certain principles should be followed. Evaluation principle plays a key role in ensuring the validity and reliability of evaluation results, and is the basic requirement of evaluation work. The evaluation index system of instructional quality designed in this paper follows the following basic principles: ① the principle of comprehensiveness, ② principle of directionality. ③ scientific principle, ④ principle of measurability, ⑤ principle of encouragement and improvement, ⑥ principle of objectivity, ⑦ principle of subjectivity, and ⑧ principle of consistency. In this paper, the teaching process, teaching environment, teaching teachers, and instructional quality monitoring are the first-level indicators of the instructional quality evaluation index system. There are several secondary indicators under the primary indicators. The evaluation index system of instructional quality constructed in this paper is shown in Table 1.

After the establishment of the evaluation index system, it is more scientific to set the corresponding weights of the index system. In the process of setting weights, we can first establish the weights set by experts' evaluation indicators and build a BPNN model learning network through the powerful function of MATLAB toolbox according to the weights of experts' indicators. Then, we input the weights set by experts and adjust the weights of indicators set by experts through network training according to the functions of BPNN model. The final error is minimized, and the weight is relatively more reasonable. The user management unit of the system is divided into two aspects: system login and security management. Different types of users log in to the instructional quality evaluation system according to different unit entrances, and users perform operations in their respective permission pages. Considering the security of user information, users are divided into five types: teaching supervisor, audit administrator, administrator, teacher, and student.

## 4. Result Analysis and Discussion

**Figure 3:**
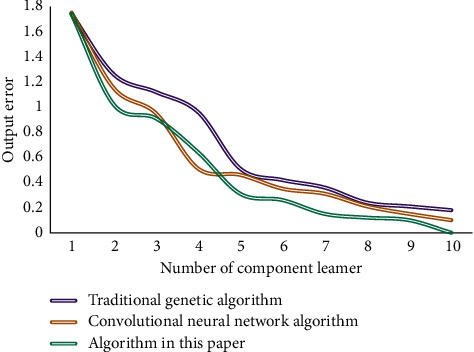
Convergence curve of the algorithm.

The instructional quality evaluation system designed based on DL algorithm in this paper contains a large amount of data, so there are certain requirements for the running hardware and software environment. In order to ensure the efficient operation of the system, the test environment in this paper is set as follows: the CPU is 2.4 GHz, the memory size is 8 GB, the computer hard disk is 1 T, and the system is Windows. In this paper, the number of nodes in the input layer, hidden layer, and output layer is 16 × 4 × 1, respectively; the learning rate is 0.8; and the function of each node adopts the Sigmoid function. After testing, the convergence curve of the algorithm is obtained as shown in Figure 3.

**Figure 4:**
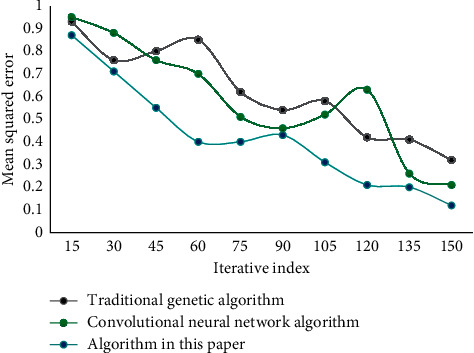
Comparison of mean square error of different algorithms.

**Figure 5:**
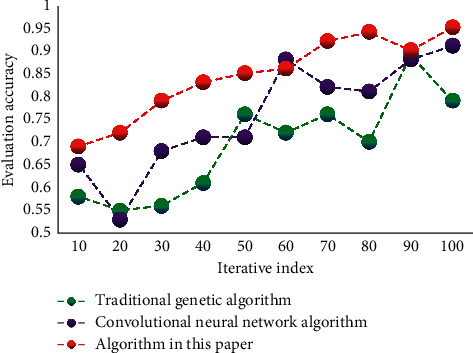
Comparison of accuracy of different algorithms.

The commonly used mean square error and accuracy rate are selected as the indexes to test the performance of this algorithm. These two indexes are used in different algorithms for testing and comparison. The mean square error of different algorithms is shown in Figure 4. The accuracy of different algorithms is shown in Figure 5.

The results show that this algorithm can not only speed up the convergence of the network but also improve the evaluation accuracy of the model. Its performance is better than that of the comparison model. Taking ten math classes in the second grade of a school as examples, the quality of math teaching was evaluated, respectively. The evaluation results of mathematics instructional quality are shown in Table 2.

**Figure 6:**
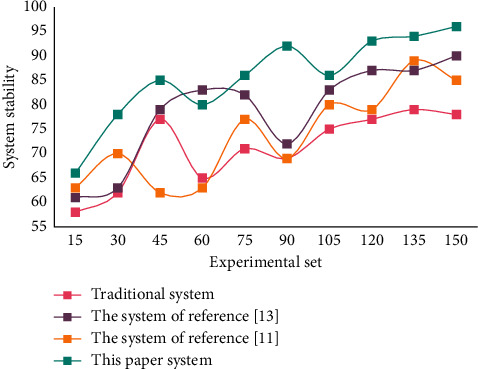
System stability test results.

The above statistics of mathematics instructional quality evaluation in different classes show that this system can identify the highest score, lowest score, and average score of instructional quality evaluation in different classes. It provides different types of data for comprehensive analysis of mathematics instructional quality.  Figure 6 shows the stability test results of different systems.

**Figure 7:**
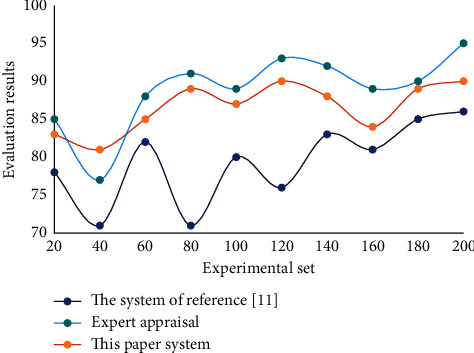
Comparison between different system evaluation results and expert evaluation results.

It can be seen from Figure 6 that the stability of this system is high. Moreover, the analysis and suggestion function of the instructional quality evaluation system designed in this paper reflects the degree of intelligence of the system, shares the workload of manually evaluating teachers' teaching effect, and improves the operational efficiency of instructional quality evaluation. Through training, quantifiable classification criteria or experts' experience information that is not easy to quantify is stored in the nonlinear network, and the instructional quality evaluation NN is measured with new experimental data. Comparison between different system evaluation results and expert evaluation results is shown in Figure 7.

It can be seen from Figure 7 that, compared with other systems, the evaluation network constructed in this paper can give results consistent with expert evaluation. This shows that this method has certain accuracy and reliability. The experimental results show that the stability of this system is high, reaching 96.37%, and the evaluation accuracy rate can reach 95.42%. This system has certain reliability. Compared with the general instructional quality evaluation system, the instructional quality evaluation system based on DL algorithm has the advantage of being able to analyze the existing problems in teaching and put forward suggestions for improving instructional quality.

## 5. Conclusions

With the continuous improvement of teaching levels and the rapid development of classroom instructional mode, the optimization of mathematics classroom instructional mode has become increasingly important in the process of instructional mode innovation. Mathematical instructional mode optimization has a distinct value and function. Teachers must update their teaching concepts, adopt various teaching strategies to promote the development of students' mathematical thinking, and develop students' thinking flexibility and creativity through the training of various mathematical exercises in order to continually optimize mathematics instructional mode and construct more efficient classroom teaching. Simultaneously, mathematics teachers should have a thorough understanding of how to optimize classroom teaching content in order to effectively improve the overall level of mathematics teaching through practice. The current state of mathematics instructional mode is examined in this paper, as well as the implementation strategy for mathematics classroom teaching optimization. Furthermore, the instructional quality evaluation system is based on the DL algorithm. This paper's system has a high level of intelligence. It can accurately assess instructional quality while also assessing problems in the teaching process based on the instructional quality scores and making reasonable suggestions for teaching improvement based on the weak links in the teaching process. The experimental results show that the system's stability is high, with a 96.37 percent stability rate and a 95.42 percent evaluation accuracy rate. This system has a high level of reliability and practical utility, and it can be used to develop a feasible scheme for evaluating instructional quality. The analysis and suggestion function of the instructional quality evaluation system proposed in this paper reflects the system's intelligence, reduces the workload of manually evaluating teachers' teaching effectiveness, and improves the operational efficiency of instructional quality evaluation. However, due to my limited knowledge and time, there are still some flaws in this paper's instructional quality evaluation system. The next step will be to discuss how to improve the system's efficiency without sacrificing accuracy.

## Figures and Tables

**Table 1:** Instructional quality evaluation index system.

Instructional quality evaluation index	Primary index	Secondary index	Index sequence
	Teaching process	Degree of interaction	X1
		Assess rationality	X2
		Organizational science	X3
		Cultivation of innovative ability	X4
		Cultivation of practical ability	X5
		Cultivating students' interest	X6
		Students' final grades	X7
	Teaching teachers	Scientific teaching	X8
		Teaching attitude	X9
		Teaching ability	X10
		Teach students in accordance with their aptitude	X11
	Teaching environment	Supporting facilities	X12
		Practice frequency	X13
	Instructional quality monitoring	Teacher self-evaluation	X14
		Student evaluation	X15
		Teachers' mutual evaluation	X16
		Leadership evaluation	X17
		Teaching supervision	X18

**Table 2:** Evaluation results of mathematics instructional quality of ten classes.

Classes	Lowest score	Highest score	Average score
1	89.6	92.7	91.2
2	91.5	94.5	93.1
3	88.3	92.6	90.5
4	92.1	95.8	94.2
5	89.3	94.5	92.5
6	88.9	93.1	90.8
7	89.6	93.5	91.7
8	84.7	89.8	86.6
9	86.1	90.5	88.2
10	89.4	92.7	90.9

## Data Availability

The data used to support the findings of this study are available from the author upon request.
